# Characterization and Rheological Properties of a New Exopolysaccharide Overproduced by *Rhizobium* sp. L01

**DOI:** 10.3390/polym17050592

**Published:** 2025-02-23

**Authors:** Haolin Huang, Yaolan Wen, Zhuangzhuang Li, Biao Wang, Shuang Li

**Affiliations:** 1 College of Biotechnology and Pharmaceutical Engineering, Nanjing Tech University, Nanjing 211816, China; 202262118011@njtech.edu.cn (H.H.); 202261218125@njtech.edu.cn (Y.W.); 2 Petroleum Engineering Technology Research Institute of Sinopec Jiangsu Oilfield Company, 188 Weiyang Road, Yangzhou 225000, China; lizz.jsyt@sinopec.com (Z.L.); wangbiao.jsyt@sinopec.com (B.W.)

**Keywords:** *Rhizobium*, *Rhizobium tropici*, exopolysaccharide, rheological properties, bioemulsifier

## Abstract

The exopolysaccharides produced by rhizobia play an important role in their biotechnological and bioremediation properties. The characteristics and properties of an exopolysaccharide produced by *Rhizobium* sp. L01 were investigated. Strain *Rhizobium* sp. L01 was identified as *Rhizobium tropici* and produced a high yield of exopolysaccharides (REPS-L01), reaching 22.8 g/L after 63 h of fermentation in a 5 L bioreactor with glucose as the carbon source. REPS-L01 was composed of glucose and galactose in a ratio of 2.95:1, carrying pyruvate, acetate, and succinate groups. REPS-L01 had good shear-thinning properties in aqueous solutions at various concentrations and revealed typical non-crosslinked polymer properties. REPS-L01 revealed thermal stability up to 275 °C. REPS-L01 had the potential to be thicker, being suitable for use under conditions ranging from 4 to 60 °C, pH between 2 and 12, and salt concentrations up to 20,000 mg/L. REPS-L01 showed strong emulsifying activity, particularly with *n*-hexane; even at concentrations as low as 0.25 wt%, the emulsification index could reach more than 50%. Even more impressively, stable *n*-hexane emulsion gel was formed with 2 wt% REPS-L01 solution. Rheological studies showed that the solid-like emulsion gel had a high storage modulus, and the SEM studies of the emulsion gel indicated that *n*-hexane could fill the pores of REPS-L01.

## 1. Introduction

Exopolysaccharide (EPS) from nitrogen-fixing rhizobial bacteria is a kind of bacterial EPS, and has the potential for industrial applications. Rhizobial exopolysaccharide (REPS) has environmental remediation properties and plays a key role in adapting to environmental stress and establishing effective legume–rhizobia symbiosis [1,2]. Compared with plant- and algae-extracted polysaccharides, bacterial EPS has the advantage of being cultured under adjustable conditions to quickly and effectively obtain the same kinds of polysaccharides with enhanced yield and controllable composition [3]. Some bacterial polysaccharides, such as xanthan, gellan, and pullulan, have been industrialized and widely used, and have also been deeply studied. However, in the case of REPS, relatively few reports on production strains or chemical and structural characteristics have been published. The yield of some efficient REPS-producing rhizobia, for instance *Sinorhizobium meliloti*, *Rhizobium radiobacter,* and *Rhizobium pusense* [4,5,6], can reach more than 20 g/L. Due to their unique rheological properties, these REPSs have potential applications as thickeners and emulsifiers in the cosmetic and food industries, and have the potential to expand to biomedical fields such as tissue engineering, regenerative medicine, and pharmaceuticals in the near future [7].

Among the EPS-producing rhizobia, *Rhizobium tropici* is well known for its potential biotechnological and bioremediation properties [8]. The type strains of *Rhizobium tropici,* such as SEMIA 4080 and SEMIA 4077, are important commercial plant-growth-promoting bacteria [9,10]. The REPSs produced by *Rhizobium tropici* SEMIA 4080 and SEMIA 4077 play an important role in plant probiotics, improving soil structures and mitigating soil erosion, increasing the number and population of soil microorganisms, and promoting root nodulation and the growth of legumes [11]. However, the yield of REPS produced by *Rhizobium tropici* reported at present is less than 10 g/L [12], which greatly limits the industrialization and application of this kind of REPS.

Therefore, the development of efficient REPS-producing strains and the characterization of their structural components and rheological characteristics are helpful to promote the application of these polysaccharides. In this study, a novel exopolysaccharide (REPS-L01) produced by the high-yield strain *Rhizobium tropici* L01 was investigated in detail for its potential functional properties. We found that REPS-L01 had excellent thickening and emulsifying properties, as found in other REPSs.

## 2. Materials and Methods

### 2.1. Isolation and Identification of Strain L01

*Rhizobium* sp. L01 was isolated from the plant rhizosphere. The genomic DNA of *Rhizobium* sp. L01 was prepared using a genomic DNA purification kit. The general bacterial primers 27F (5′-AGAGTTTGATCMTGGCTCAG-3′) and 1492R (5′-TACGGCTACCTTGTACGACTT-3′) were used to amplify the 16S rDNA gene. The PCR products were purified and sequenced with GenScript (Nanjing, China), and the obtained sequences were analyzed using the nucleotide module of the Basic Local Alignment Search Tool (BLASTN) on the National Center for Biotechnology Information (NCBI) website. L01 was cultured on LB agar plates supplemented with 20 g/L of glucose at 30 °C for 3 days to observe the colony morphology.

### 2.2. Production and Preparation of REPS-L01

The seed medium contained 20 g/L glucose, 1 g/L yeast extract, 4 g/L peptone, 2 g/L K_2_HPO_4_·3H_2_O, and 0.1 g/L MgSO_4_·7H_2_O at pH 7.0–7.2. The optimized fermentation medium comprised 50 g/L glucose, 1 g/L yeast extract, 2 g/L (NH_4_)_2_HPO_4_, and 0.1 g/L MgSO_4_·7H_2_O. The initial pH was adjusted to 7.0–7.2.

*Rhizobium* sp. L01 was first inoculated into a 250 mL volumetric flask containing 50 mL seed medium and cultured continuously for 24 h at 30 °C. The batch production of REPS-L01 was carried out in a 5 L stirred-tank bioreactor (ALPHA, Suzhou, China) at 30 °C. The seed culture (300 mL) was inoculated into the fermenter containing 3 L fermentation medium. The aeration rate was maintained at the level of 1 vvm and the control of agitation speed varied from 300 to 800 rpm. During the fermentation process, the pH of the fermentation broth was automatically controlled at 7.0 with 3M NaOH solution. The apparent viscosity of the fermentation broth was measured at 30 °C with a rotational viscometer (IKA, Staufenim, German) at 30 rpm with spindle 3.

### 2.3. Extraction and Purification of REPS-L01

REPS-L01 was extracted by alcohol precipitation, which is commonly used in polysaccharide extraction [13]. Firstly, the fermentation broth was diluted 1:1 with distilled water and put into a 75 °C water bath for 30 min. Then, the processed fermentation liquid was re-centrifuged three times to remove the cells thoroughly. After concentration, 3 times the volume of 95% ethanol was added, and the sample was put into a 4 °C refrigerator overnight and centrifuged at 8000 rpm for 20 min. The centrifuged precipitate was freeze-dried until the weight remained constant to obtain the crude polysaccharide product.

Then, we identified the proper amount of crude products and complete swelling in a 75 °C water bath and added an equal volume of Sevage reagent (CHCl_3_:BuOH = 5:1, *v*/*v*) to remove the protein in the solution [14]. We repeated this step 2–3 times until no protein precipitate was observed. The supernatant after protein removal was collected and placed in a 10,000 Da dialysis bag for dialysis and freeze-drying to obtain purified REPS-L01.

### 2.4. Analysis of REPS-L01’s Physical Properties

#### 2.4.1. Chemical Composition

The quantitative detection of chemical components, such as total sugars, proteins, and uronic acids, helps to evaluate the purity and characteristic components of polysaccharide samples. The total sugar was determined with the H_2_SO_4_–phenol method [15]. The color reaction between H_2_SO_4_–phenol and hexose in the polysaccharide was obtained at 490 nm. The maximum absorption peak and the absorption value are measured in a linear curve with the sugar content.

The uronic acid was determined with the Carbazole reaction [16]. The hexuronic acid in the sample reacts with concentrated sulfuric acid to form chromogen, which is quantitatively colored with carbazole. Glucuronic acid was used as a standard substance.

The protein content in the purified REPS-L01 was determined with the Bradford method [17]. The protein in the sample can be quantitatively bound to Coomassie brilliant blue G-250, with a maximum absorption peak at 595 nm.

#### 2.4.2. Fourier-Transform Infrared (FTIR) Spectroscopic Analysis

FT-IR analysis of purified REPS-L01 was carried out using a Tensor 27 infrared spectrophotometer (Bruker, Billerica, MA, USA). Spectra were determined between 4000 and 625 cm^−1^ at a resolution of 0.5 cm^−1^ using 10 scans.

#### 2.4.3. Molecular Weight (M_w_)

The M_w_ (weight-average molar mass), M_n_ (number-average molecular weight) and molecular mass dispersity (M_w_/M_n_) of filtered pure REPS-L01 (3 mg/mL) were analyzed using Agilent 1260 Infinity GPC/SEC MDS (a system optimized for organic use with refractive index detector, dual-angle light scattering detector, and viscometer options) (Agilent Technologies, Santa Clara, CA, USA). The column used was a PL aquagel-OH Mixed-H column (8 μm, 7.5 × 300 mm, molecular weight range 500–10,000,000). The detection conditions were set as 45 °C, 1.0 mL/min, and a sample size of 50 μL. Isocratic elution was carried out using 0.1 M NaNO_3_ (0.01% NaN_3_) as the mobile phase. The molecular weights were computed by fitting the viscosity and peak time with Agilent GPC/SEC software A.02.02.

#### 2.4.4. Monosaccharide Composition Analysis

The monosaccharide composition was measured using trifluoroacetic acid (TFA) hydrolysis, followed by IC analysis [18]. The precise weight of 10 mg of purified REPS-L01 was placed in an ampoule; then, 10 mL of 2 M TFA was added and the sample was further hydrolyzed at 120 °C for 3 h. A certain amount of acid hydrolysis solution was dried with nitrogen, 5 mL of water was added for vortex mixing, and the supernatant was analyzed by IC after centrifugation. The samples were loaded on an ICS5000 system (ThermoFisher, Waltham, MA, USA) equipped with a DionexCarbopac^TM^PA20 column at 30 °C. The monosaccharide composition of REPS-L01 could be obtained by comparing the retention time and peak area with the response of the standard (fucose, rhamnose, arabinose, galactose, glucose, xylose, mannose, fructose, ribose, galacturonic acid, glucuronic acid, aminogalactose hydrochloride, glucosamine hydrochloride, N-acetyl-D-glucosamine, guluronic acid, and mannuronic acid).

#### 2.4.5. Determination of Substituent Content

The purified REPS-L01 was fully dissolved in 0.1 M of H_2_SO_4_ solution to obtain 5 mg/mL of aqueous solution and incubated at 80 °C for 16 h to hydrolyze the substituents. And then, the REPS-L01 solution was filtered with a 0.22 μm filter membrane and the content of the REPS-L01 substituents was determined with the Alliance 2695 system (Waters, Milford, CT, USA). The analytical column used was a BIO-RAD-Aminex HPX-87H column. The mobile phase consisted of 5 mM of H_2_SO_4_. Elution was carried out at a flow rate of 0.5 mL/min at 50 °C.

### 2.5. Thermal Gravimetric Analysis (TGA-DTG)

TGA was measured with an integrated thermal analyzer (Henven, Beijing, China) under nitrogen gas. The REPS-L01 samples (10 mg) were measured from 20 to 600 °C at a rate of 10 °C/min.

### 2.6. Rheological Properties

REPS-L01 was dissolved and completely swollen in ultrapure water and configured into aqueous solutions of different concentrations (0.1, 0.25, 0.5, 0.75, and 1.0 wt%). The apparent viscosity of the above samples was measured with a rheometer (DHR-2, TA Instruments, New Castle, DE, USA) with a 25 mm parallel plate fixture. All tests were performed at 25 °C. The dynamic frequency behavior was measured in the range of 0.01–10 Hz at 1% strain. Viscosity data were collected at shear rates from 0.01 s^−1^ to 100 s^−1^. Temperature sweeps were carried out in the range of 25–85 °C at 1% strain, and Origin was used to process the data (OriginLab, Northampton, MA, USA).

### 2.7. Viscosity Tests

#### 2.7.1. Effects of Temperature on Apparent Viscosity

The apparent viscosity of the REPS-L01 solutions (0.5 wt%) was measured at 4 °C, 20 °C, 40 °C, 60 °C, and 80 °C. Samples treated at 4–60 °C were measured on a rotational viscometer at 30 rpm with spindle 3, as described in Section 2.2. Samples treated at 70–100 °C were measured on a rotational viscometer at 30 rpm with spindle 1.

#### 2.7.2. Effects of pH on Apparent Viscosity

The pH of the REPS-L01 solutions (0.5 wt%) was adjusted to 2, 4, 6, 8, 10, and 12 with HCl (0.1 M) or NaOH (0.1 M). All samples were measured on a rotational viscometer at 25 °C, as described in Section 2.2.

#### 2.7.3. Effects of Salinity on Apparent Viscosity

The brine water contained total salinities of 2000, 5000, 10,000, and 20,000 mg/L, respectively, as described in Table 1. The REPS-L01 samples were fully swollen in brine water to prepare the solutions (0.5 wt%). All samples were measured on a rotational viscometer, as described in Section 2.2.

### 2.8. Emulsification Index (E_24_)

Diesel, soybean oil, liquid paraffin, xylene, n-hexane, *n*-octane, dodecane, and tetradecane were selected as the substrates. REPS-L01 solution and substrate were mixed in a ratio of 1:1. After shaking and mixing, the height of the emulsion layer and water was determined for 24 h at 30 °C. The emulsification index (E_24_) was calculated according to the following formula:

(1) $$E_{24}=(he/ht)\times 100\%$$

where *he* is the height of the emulsion layer and *ht* is the height of the total mixture.

### 2.9. Scanning Electron Microscopy (SEM) Studies

The samples were quenched in liquid nitrogen at room temperature and then freeze-dried into powder [19]. The sample powders were sprayed with gold, and the prepared samples were glued to the sample table [20]. These samples were observed using a field-emission scanning electron microscope (ThermoFisher, Massachusetts, USA) at an accelerating voltage of 30 kV. Images with magnifications of 200× and 500× were recorded.

## 3. Results and Discussion

### 3.1. Identification of Rhizobium sp. L01

**Figure 1:**
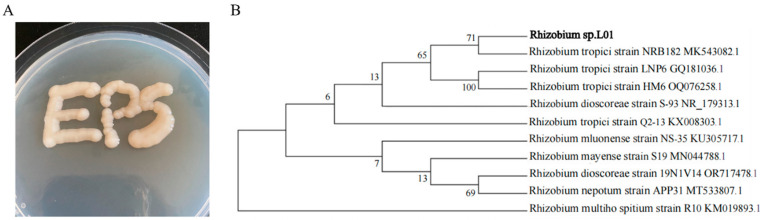
(**A**) The colony morphology of strain L01. (**B**) Phylogenetic analysis of the L01 isolate based on the sequencing of 16S rDNA.

Strain L01 was isolated from the rhizosphere soil. The colony morphology of strain L01 formed a smooth, mucoidal, round shape when grown with glucose as the carbon source (Figure 1A), indicating its ability to overproduce exopolysaccharides. BLAST analysis showed that the 16S rDNA sequence of strain L01 had the highest identity (99%) with *Rhizobium tropici* (Figure 1B). Therefore, strain L01 was named *Rhizobium* sp. L01, and has been deposited at the China Center for Type Culture Collection (CCTCC NO: 20222005). The *Rhizobium* sp. L01 sequences used in this study are publicly available at the NCBI Sequence Read Archive (SRA) under accession ID SRR29533191.

### 3.2. Production of REPS-L01

**Figure 2:**
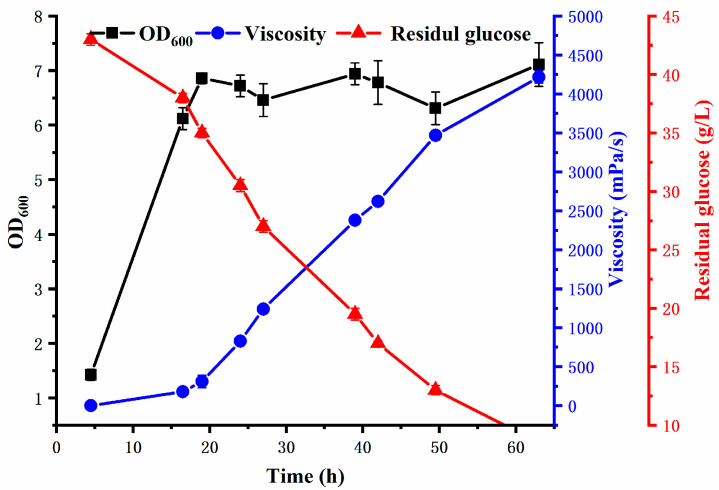
Time course of REPS-L01 production by the strain *Rhizobium* sp. L01 in optimized fermentation process.

Strain L01 could efficiently produce the polysaccharide REPS-L01 using glucose as a carbon source and ammonium salt as a nitrogen source in a 5 L fermenter, as shown in Figure 2. Similar to many rhizobia reported in the literature, strain L01 produced fast-growing and high-yielding polysaccharides with minimum nutritional requirements, making it a potential cell factory for EPS production. The growth of bacteria was measured by cell density (OD_600nm_). Rapid growth of the bacteria occurred in the first 14 h, and then microbial growth entered a stationary phase, at which point the EPS began to accumulate, and the fermentation broth became viscous after 20 h of fermentation. After fermenting for 63 h, glucose was almost exhausted, the apparent viscosity of the fermentation liquid was 4200 ± 80 mPa·s, and the yield of REPS-L01 was about 22.8 g/L.

Current research indicates that the maximum yield of *Rhizobium tropici* strains is 7.45 g/L [8], while the average yield of *Rhizobium pusense* strains has reached 20 g/L [5]. *Rhizobium radiobacter* strain ATCC 18052 N-11 was found to have the highest exopolysaccharide production, achieving a yield of 32.5 g/L in a 15 L fermenter [4]. Notably, the yield of *Rhizobium tropici* strain L01 in our study reached 22.8 g/L when fermented in a 5 L fermenter, which was very impressive. Furthermore, glucose was used as the carbon source for REPS-L01 production, which is much cheaper than sucrose, resulting in a relatively low production cost of REPS-L01. Moreover, the fermentation period of REPS-L01 was only 63 h, which is much shorter than the current REPS fermentation time of 72 h [4] or 96 h [12]. This indicates that REPS-L01 has high production efficiency and good industrial prospects.

### 3.3. Determination of Monosaccharide Composition and Substituent Groups

The polysaccharide produced from the fermentation of *Rhizobium* sp. L01 was extracted and purified by alcohol precipitation, deproteinization, dialysis, and lyophilization. The purified REPS-L01 polysaccharide was obtained in the form of an off-white feathery powder. The total sugar, uronic acid, and protein contents of REPS-L01 are shown in Table 2. The molecular weight distribution of REPS-L01 is shown in Figure S1. The M_w_ and M_n_ values of REPS-L01 were 3.04 × 10^6^ g/mol and 2.63 × 10^6^ g/mol, respectively, which are consistent with the molecular weight distribution (1.55 to 5.30 × 10^6^ g/mol) of most REPSs confirmed in the literature [21]. The dispersity index (M_w_/M_n_) of REPS-L01 was 1.155, which is close to 1.0, indicating that REPS-L01 is a homogeneous polysaccharide [22]. Also, this index is related to the rheological properties of polymers: samples with a lower dispersion index are less sensitive to shear, and the weight of viscous deformation in the total deformation is lower [23].

Monosaccharide composition is an important characteristic parameter of polysaccharide structure, and variations in monosaccharide type and proportion directly lead to differences in polysaccharide structure. The monosaccharide composition analysis of REPS-L01 and a standard sample are shown in Figures S2 and S3. These indicate that REPS-L01 contained only glucose and galactose, with a molar ratio of about 2.95:1. Similarly to most polysaccharides synthesized by rhizobial strains, the repeating sugar units were mainly composed of glucose and galactose (Table 3). The molar ratio of glucose to galactose of succinylglycan was 7:1 [7], and the REPSs from both strains of *Rhizobium pusense* KM7 and *Sinorhizobium meliloti* SMC1 fit the typical compositional profile of succinylglycan. The molar ratio of glucose to galactose of the REPSs produced by the *Rhizobium tropici* strains SEMIA 4077, 4080, and MUTZC3 were similar, ranging from 1.3:1 to 1.9:1. Unlike the REPSs produced by the strains SEMIA 4077 and 4080, REPS-L01 contained no glucuronic acid or galacturonic acid in its repeat units. The molar ratio of glucose to galactose of REPS-L01 was similar to that of the polysaccharide produced by *Rhizobium tropici* strain SRA1, approaching 3:1.

The quantitative analysis of non-carbohydrate substitution by HPLC showed that REPS-L01 was substituted with significant amounts of pyruvic, acetic, and succinic groups, accounting for 66.1 mg/g, 28.3 mg/g, and 10.2 mg/g, respectively. The types of organic substitutes are widely existed in REPSs [24] and the contents of organic substitutes could be related to the characteristics of producer strains and be influenced by certain environments [25,26,27,28]. Although uronic acid was not present in REPS-L01, the presence of succinic groups made it anionic, promoting the emulsifying and rheological properties of the polysaccharides [29].

**Table 3:** Monosaccharide composition of the exopolysaccharides produced by rhizobial strains.

Strains	Monomer Composition (%)						Substituent	Yield (g/L)	Reference
	Glc	Gal	GlcA	GalA	Man	Rha			
*Rhizobium* sp. L01	74.7	25.3	nd	nd	nd	nd	Succinate, pyruvate, and acetate	22.8	This work
*Rhizobium tropici* SEMIA 4077	59.59	31.29	4.58	0.08	0.74	3.72	Acetate, carboxy	7.45	[8]
*Rhizobium tropici* SEMIA 4080	55.48	32.47	8.6	tr	0.86	2.58	Succinate, pyruvate, and acetate	2.52	[30]
*Rhizobium tropici* MUTZC3	53.53	40.42	tr	2.60	0.74	2.60	Succinate, pyruvate, and acetate	4.39	[30]
*Rhizobium tropici* LBMP-C01	314.7 μg/mL	30.5 μg/mL	-	1.0 μg/mL	2.0 μg/mL	2.0 μg/mL	Hydroxyl and carboxyl	3.97	[31]
*Rhizobium tropici* SRA1	In a molar ratio of 3:1		-	-	-	-	Hydroxyl, carboxyl, and methoxyl	0.55	[32]
*Rhizobium viscosum* CECT908	35.0	43.7	17.1		2.0	-	O-acetyl	6.1	[33]
*Rhizobium pusense* ZB01	100	-	-	-	-	-	O-acetyl	3.18	[34]
*Rhizobium pusense* KM7	85.3	12.2	-	-	-	-	Hydroxyl, carboxyl, and succinyl	21	[5]
*Sinorhizobium meliloti* SMC1	In a molar ratio of 7:1		-	-	-	-	Succinate, pyruvate, and acetate	22.3	[6]
*Rhizobium radiobacter* ATCC 18052 N-11	85.59	12.92	-	-	0.94	-	Succinate, pyruvate, and acetate	32.5	[4]

Man, mannose; Rha, rhamnose; GlcA, glucuronic acid; GalA, galacturonic acid; Glc, glucose; Gal, galactose; tr, trace; nd, not detected.

### 3.4. Fourier-Transform Infrared (FTIR) Spectroscopic Analysis

**Figure 3:**
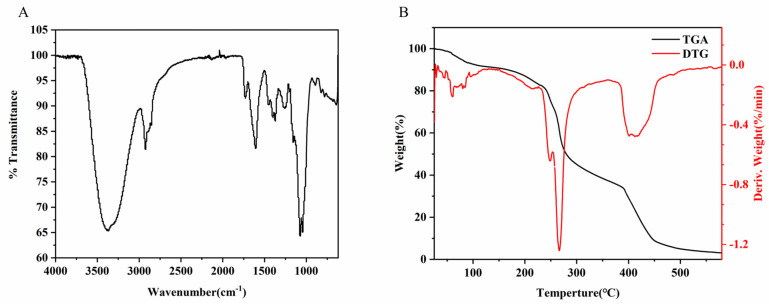
(**A**) FTIR spectra of REPS-L01. (**B**) TGA and DTG curves of REPS-L01 from 20 to 600 °C.

The structure of REPS-L01 was analyzed using FTIR. The spectra are shown in Figure 3A. The broad absorption peak at 3373 cm^−1^ was caused by the O-H stretching. The peak at 2923 cm^−1^ was caused by the asymmetric CH vibrations in -CH_2_ and -CH_3_. The peaks at 1732 cm^−1^ and 1606 cm^−1^ were caused by the asymmetric C=O stretching vibrations of the acetyl and acetone groups, respectively. The absorption peak at 1373 cm^−1^ was caused by the symmetric stretching vibration of the carboxylate -COO- group from the acid residues. At the same time, the strong absorption peak at 1606 cm^−1^ also proved that the content of pyruvic acid in REPS-L01 was high, which is consistent with the results of the HPLC analysis. In addition, the carbohydrates showed C-O vibration at 1074 cm^−1^ and the sugar backbone showed asymmetric C-O-C stretching at 1045 cm^−1^. All these absorption patterns are very similar to those found in typical microbial EPSs, as reported previously in the literature [35,36].

### 3.5. Thermal Analysis

To evaluate the potential application of REPS-L01 in industry, its thermal stability was tested. Increasing temperatures are often accompanied by endothermic and exothermic states, resulting in crystal melting and structural destruction [37]. The thermogravimetric curve and differential thermal gravimetry curve shown in Figure 3B indicate that REPS-L01 changed in three stages during the heating process. In the range of 20 to 180 °C, the mass of REPS-L01 decreased (−11%), largely due to the desorption of moisture from the sample surface and the bulk as a result of the breaking of hydrogen bonds between water molecules and polar functional groups [38]. The differential thermal gravimetry curve had three pronounced peaks at 248, 262, and 397 °C, which were divided into two stages. The first degradation occurred in the range of 180–280 °C, and when the temperature rose to 262 °C, the weight of EPS-L01 dropped sharply, which was related to the degradation of the main chain structure of the EPS [39]. The second stage occurred at about 390 °C and terminated at 450 °C with about 91% of REPS-L01 degraded, which might be due to the decomposition of macromolecular or inorganic impurities [40]. The final residue was found at 3.09%, which might be due to the stable byproducts obtained from degradation. The results showed that REPS-L01 had good relative thermal stability below 275 °C.

### 3.6. Rheological Properties of REPS-L01

#### 3.6.1. Steady Shear Measurement

**Figure 4:**
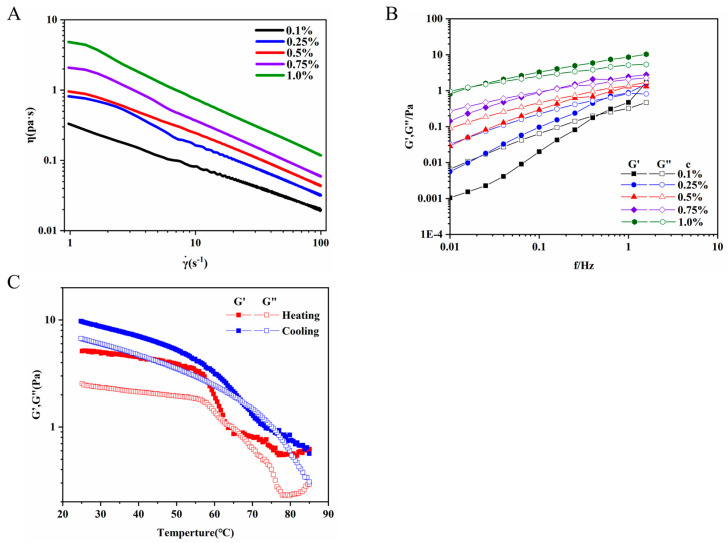
(**A**) Steady shear curve of REPS-L01. (**B**) Dynamic viscoelastic curve of REPS-L01. (**C**) Temperature sweep curve of REPS-L01.

In viscosity tests, several factors—such as molecular weight, intermolecular interactions, conformations, and substituents—affect the viscosity of EPSs [41]. Furthermore, we studied the rheological properties of REPS-L01. The steady-state shear of REPS-L01 was identified in the range of concentration of 0.1–1.0 wt% (Figure 4A), and shear rates ranged from 1 to 100 s^−1^. The viscosity of the REPS-L01 aqueous solution increased with increasing concentration and showed pseudoplastic behavior, as is the case with other REPSs such as succinoglycan [4]. This indicated that REPS-L01 is concentration-dependent and has the potential to be used as a thickener at different concentrations. The viscosity of the REPS-L01 aqueous solution decreased sharply with concentration, showing typical random coil polysaccharides [42]. The apparent viscosity of the fluid decreases with increases in shear rate, which is known as the shear thinning phenomenon. This characteristic is very important in industrial production, and means that the REPS-L01 solution can flow easily when poured from a container or during certain processing operations, such as pumping, spray-drying, or stirring, despite its high viscosity [43].

#### 3.6.2. Dynamic Viscoelastic Behavior of REPS-L01

The results of the dynamic frequency sweep tests of the 0.1–1.0 wt% REPS-L01 solutions are shown in Figure 4B. Elastic modulus (G′) is a measure of resistance to elastic deformation, reflecting the degree of structuring in a sample, and viscous modulus (G″) is a measure of the resistance to liquid flow, reflecting the viscosity characteristics of a test sample [44]. At various concentrations, both the viscous modulus (G″) and the elastic modulus (G′) increased with increasing frequency. With the increase in concentration, the REPS-L01 solution changed from being mainly viscous (G″ > G′) to mainly elastic (G″ < G′), which may be explained by the rise in the solution’s relaxation time due to the increased density of macromolecular entanglements [45]. Both the G′ and G″ of the REPS-L01 solution showed significant dependency on frequency. In the low-frequency region of 0.01 Hz to 0.1 Hz, the elastic modulus increased significantly with the increase in REPS-L01 concentration, indicating that a large amount of entanglement points and mechanical networks between polysaccharide chains and adjacent chains were formed [46]. With increasing frequency, the REPS-L01 solution gradually changed from a liquid state at low frequencies (G″ > G′) to a semi-solid state (G″ = G′), and finally to a solid-like state at high frequencies (G″ < G′). In other words, REPS-L01 exhibited sol properties at low frequencies, and with increases in frequency, the chains of REPS-L01 formed a temporary network structure; high concentrations of REPS-L01(0.75–1.0 wt%) showed weak gel-like properties in the high frequency region of 0.1 Hz to 1 Hz. The viscoelastic properties of REPS-L01 make it potentially applicable in thickening and gelation.

#### 3.6.3. Temperature Sweeps

In order to evaluate the REPS-L01 solution’s stability, temperature evolution measurements were evaluated to monitor G′ and G″ in 1.0 wt% REPS-L01. The developments of G′ and G″ during cooling and heating are shown in Figure 4C. At the initial heating stage (from 25 °C to 56 °C), the modulus of REPS-L01 decreased slightly, with G′ being higher than G″, and the solution showed a solid-like nature. Starting at 56 °C, both moduli dropped sharply, and the intersection point was observed at 67 °C, signifying the onset of gel melting and the transition to a liquid-like state. When REPS-L01 was heated, both G′ and G″ decreased, indicating that high temperature reduces the solution’s relaxation time [45]. During the cooling process, both the G′ and G″ of REPS-L01 increased with decreasing temperature, suggesting that the interactions between macromolecules (such as hydrogen bonds) in the REPS-L01 system changed during cooling, leading to an increase in the viscoelasticity of the system. The temperature of the intersection in the cooling process was significantly higher than that in the heating process, which was caused by thermal hysterescence, indicating that the capacity required for the linking and decoupling of REPS-L01 is different, and REPS-L01 is thermally irreversible [47]. The results of the temperature sweeps showed that the REPS-L01 solution is suitable for practical application in medium- and low-temperature environments.

#### 3.6.4. Stability Characteristics in Different Environments

The viscosity and rheological properties of most hydrophilic EPSs in aqueous solutions determine their industrial applications, including as emulsifiers, emulsion stabilizers, thickeners, and flocculants in food, cosmetics, and other applications [48]. Information on the influence of pH, temperature, and ionic strength would be useful for industrial manufacturers in selecting the polysaccharide with the desired mechanical properties for pharmaceutical, nutraceutical, and cosmetic formulations [49]. The apparent viscosity of REPS-L01 in aqueous solution in different environments is an important polymer characteristic parameter.

**Figure 5:**
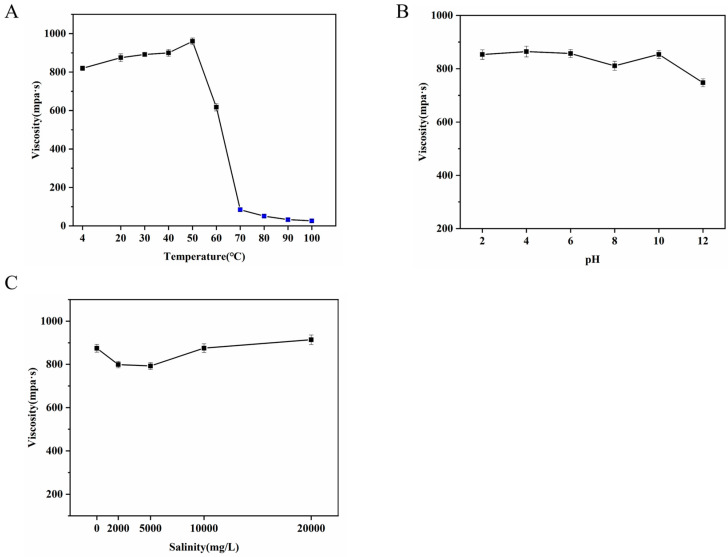
(**A**) Effect of temperature on apparent viscosity of 0.5 wt% REPS-L01 solution. (**B**) Effect of pH on apparent viscosity of 0.5 wt% REPS-L01 solution. (**C**) Effect of metal ions on apparent viscosity of 0.5 wt% REPS-L01 solution.

Changes in temperature will affect the movement of EPS molecules and thus affect their rheological properties. According to the Arrhenius model, the effect of temperature on viscosity must be considered because biopolymers may be processed at different temperatures [50]. As shown in Figure 5A, with the increase in temperature, the viscosity of the REPS-L01 solution increased slightly. However, when the temperature reached 70 °C, the viscosity of REPS-L01 decreased sharply. When the temperature reached 80 °C, the REPS-L01 solution almost completely lost its viscosity, which might be related to the weakness of the interactions between molecules caused by the increase in the thermal motion between molecules [51]. In particular, the viscosity of REPS-L01 at low temperatures (4 °C) was very similar to that at 20 °C, which indicates that REPS-L01 solution is also very stable at low temperatures, and is suitable for use at temperatures of 4–60 °C.

The viscosity of polysaccharides could be influenced by pH [52]. As shown in Figure 5B, the viscosity of the EPS-L01 solution remained stable over a wide pH range (2.0–12.0), which is consistent with the properties of succinylglycan [37]. This pH stability was likely attributable to the presence of succinic groups in the structure of REPS-L01.

The apparent viscosity of polysaccharides is influenced by metal ions such as Na^+^, K^+^, Mg^2+^, and Ca^2+^ [53], which are present in significant quantities in seawater. Consequently, we prepared brine water to investigate the impact of varying concentrations of these metal ions on the apparent viscosity of REPS-L01. As shown in Figure 5C, the presence of salts had little effect on the viscosity of the REPS-L01 solution, but low concentrations of salts made the viscosity of REPS-L01 decrease slightly, which might be due to the cations shielding the electrostatic repulsion of the charged groups of REPS-L01, leading to the shrinkage and polymerization of the structure [54]. The presence of a high concentration of salts increased the viscosity of REPS-L01, which might be due to the formation of new bonds between cations, resulting in an increase in viscosity [55].

### 3.7. The Emulsion Performance of REPS-L01

#### 3.7.1. Emulsifying Activity Toward Different Hydrocarbons

**Figure 6:**
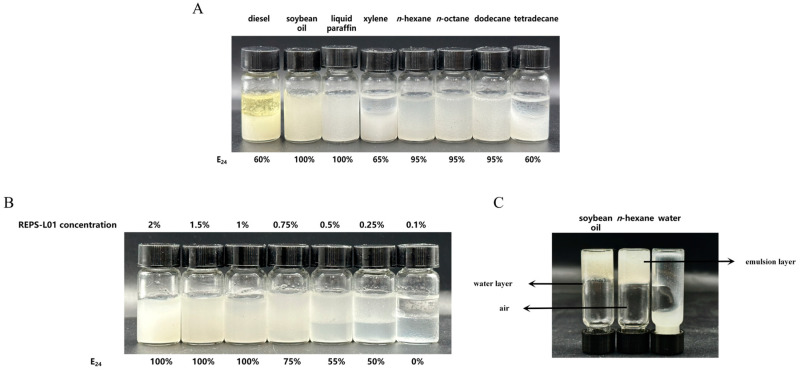
(**A**) Emulsifying activity of REPS-L01 solution (1 wt%) on different substrates. (**B**) The emulsions formed by different concentrations of REPS-L01 solution on *n*-hexane. (**C**) Gelling performance of emulsion layer: inverted vial test after mixing hydrocarbons or water with REPS-L01 solution (2 wt%).

Diesel, soybean oil, liquid paraffin, xylene, *n*-hexane, *n*-octane, dodecane, and tetradecane were selected as substrates. REPS-L01 aqueous solution (1 wt%) was mixed with the hydrocarbons at a volume ratio of 1:1, and the emulsification index E_24_ was determined after standing for 24 h at room temperature. According to the findings of Lopes, Castellane, Moretto, Lemos, and Souza [56], the criteria for the determination of emulsion-forming capacity is the ability of the emulsifier to uphold an emulsification index of at least 50% for 24 h. As shown in Figure 6A, the REPS-L01 solution exhibited good emulsifying activity toward most of the selected substrates. In particular, the E_24_ of soybean oil, liquid paraffin, *n*-hexane, *n*-octane, and dodecane reached more than 95%, showing that these hydrocarbons are the preferred substrates for the REPS-L01 solution.

High-molecular-mass bioemulsifers include amphipathic polysaccharides, proteins, lipopolysaccharides, lipoproteins, or complex mixtures of these biopolymers [57], such as Emulsan and Alasan, in which protein is considered to be the main functional substance, and polysaccharide plays the role of stabilizing the emulsion [58]. In our previous work, we found an emulsifying protein named AXE from *Bacillus subtilis* and designed bioemulsifers based on combinations of different polysaccharides with AXE. However, in this work, the protein content of purified REPS-L01 was only 0.68%, indicating that polysaccharide was the main functional substance.

#### 3.7.2. Effect of REPS-L01 Concentration on Emulsion Activity

To evaluate the influence of REPS-L01 concentration on emulsion activity, the E_24_ of the emulsions prepared with different concentrations of REPS-L01 solution (0.1, 0.25, 0.5, 0.75, 1, 1.5, and 2 wt%) and *n*-hexane at the volume ratio of 1:1 was determined. As shown in Figure 6B, when the REPS-L01 concentration exceeded 0.25 wt%, it could form a stable emulsion with an E_24_ above 50%. This indicates that REPS-L01 has high emulsifying activity and the working concentration could be as low as 0.25 wt%. Its excellent emulsifying performance means that REPS-L01 could be used as a bioemulsifier. Further, reducing the REPS-L01 concentration (0.1 wt%) led to an unstable emulsion, possibly due to the inhibition of the electrostatic interaction between the emulsifier and the substrate [56].

When the REPS-L01 concentration reached 2 wt%, the emulsification layer formed with soybean oil or *n*-hexane was tight and solid-like, and the stable emulsion did not collapse even if flipped over, as shown in Figure 6C. We call this emulsion gel. However, although the emulsification index for soybean oil was similar to *n*-hexane, a small amount of water flow could be observed in the soybean oil emulsion after flipping, which may be caused by the certain specificity of polysaccharides in forming the emulsion with hydrocarbons.

Most REPSs, especially succinoglycans, have the potential to be used as bioemulsifiers and are good candidates for application in the petroleum, environmental remediation, and agriculture industries [59]. When *n*-hexane was used as the emulsifying substrate, REPS-L01 had a similar emulsifying activity to succinoglycan CAS and SG-N, and was significantly superior to REPS SEMIA 4077, 4080, and SRA1 (Table 4). REPS-L01 is composed of glucose and galactose with succinyl modification. These characteristic components make REPS-L01 very similar to succinoglycan, but the molar ratio of glucose to galactose in REPS-L01 is close to 3:1, which is far from the 7:1 ratio of succinoglycan. Some *Rhizobium tropici* polysaccharides had a similar molar ratio of glucose to galactose (mostly 2:1 to 3:1), but could not exhibit the same efficient emulsifying properties. The relationship between the emulsifying function of polysaccharides and their structural characteristics needs to be studied further.

**Table 4:** Emulsifying activity (%) of REPS-L01 and other REPSs on different hydrophobic substances.

Strain	REPS	Substrate	Concentration	E_24_	Reference
*Rhizobium* sp. L01	L01	*n*-hexane	2%	100%	This work
			1%	95%	
			0.5%	75%	
		Soybean oil	1%	100%	
		Paraffin liquid oil	1%	100%	
*Rhizobium radiobacter* CAS	CAS	Soybean oil	1%	100%	[60]
			0.5%	94%	
		*n*-hexane	1%	100%	
			0.5%	65%	
*Rhizobium radiobacter* ATCC 18052 N-11	SG-N	Soybean oil	0.15%	85.3%	[4]
*Rhizobium tropici* SEMIA 4080	SEMIA 4080	*n*-hexane	5%	34.67%	[12]
		Paraffin liquid oil	5%	38.89%	
*Rhizobium tropici* SEMIA 4077	SEMIA 4077	Soybean oil	0.1%	48.68%	[8]
			0.5%	78.05%	
		Diesel	0.1%	1.95%	
			0.5%	51.22%	
*Rhizobium tropici* SRA1	SRA1	*n*-hexane	0.5%	73.33%	[32]
		Toluene	0.5%	76.66%	

#### 3.7.3. Rheological Properties of Emulsion Gel

**Figure 7:**
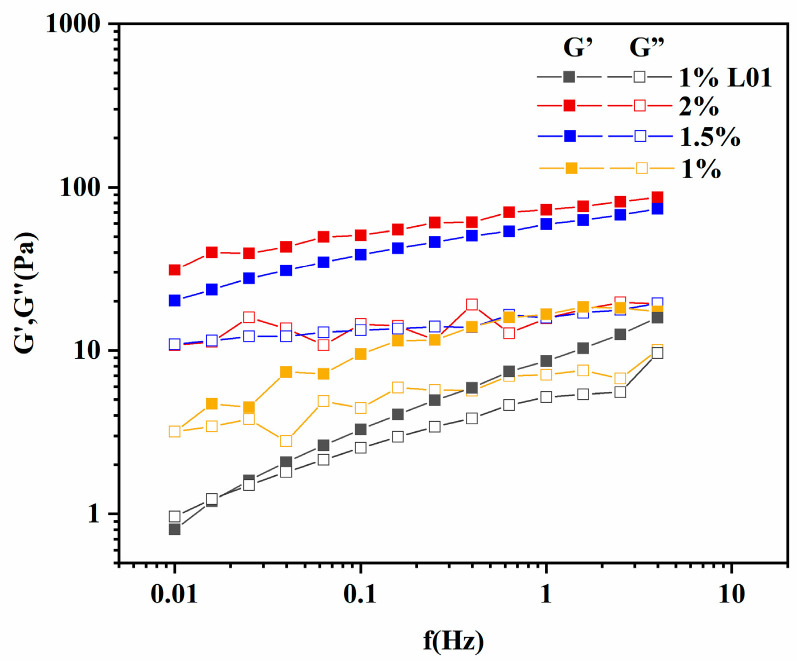
The dynamic viscoelastic properties of REPS-L01 aqueous solution (1 wt%, black) and *n*-hexane emulsion gels with different concentrations of REPS-L01 solution (1 wt%, 1.5 wt%, and 2 wt%; yellow, blue, and red).

Due to the specificity of *n*-hexane emulsion gel, the rheological properties of *n*-hexane emulsion gels formed with different concentrations of REPS-L01 solution (1, 1.5, and 2 wt%) were investigated and compared with 1 wt% REPS-L01 aqueous solution. From the frequency scanning diagram (Figure 7), 1 wt% REPS-L01 aqueous solution had a phase shift with increases in frequency, and only showed weak gelatinicity under high-frequency conditions (frequency-dependent). The emulsion gel formed by REPS-L01 and *n*-hexane showed a gel-like behavior with G′ > G″ at all frequencies, which indicated that the presence of *n*-hexane changed the rheological properties of REPS-L01. As the concentration of REPS-L01 in the emulsion gel increased (1, 2 wt%), the elastic modulus of the emulsion gel became more stable.

In recent years, the hydrogels of EPSs have attracted attention. For example, succinoglycan synthesized by *Sinorhizobium meliloti* Rm 1021 can be combined with Fe^3+^ to form hydrogels [61], and the same can be achieved with other bacterial polysaccharides, such as xanthan gum [62] and gellan gum [63]. The gelling performance of emulsions formed with EPSs and hydrocarbons was recently reported for the first time, which further enriched the gelation research on EPSs.

#### 3.7.4. Micromorphology of Emulsion Gels

**Figure 8:**
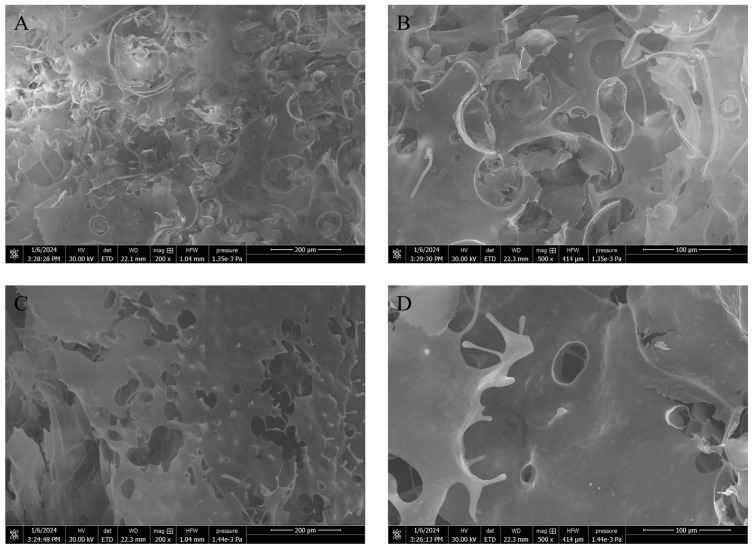
(**A**) SEM of 1 wt% REPS-L01 aqueous solution under 200× magnification. (**B**) SEM of 1 wt% REPS-L01 aqueous solution under 500× magnification. (**C**) SEM of *n*-hexane emulsion gel formed with 2 wt% REPS-L01 under 200× magnification. (**D**) SEM of *n*-hexane emulsion gel formed with 2 wt% REPS-L01 under 500× magnification.

To further study the emulsion gels, the microscopic morphology of *n*-hexane emulsion gel formed with 2 wt% REPS-L01 solution was observed with a scanning electron microscope (SEM), and 1 wt% REPS-L01 aqueous solution was used as a control due to having the same content of REPS-L01. As shown in Figure 8A,B, 1 wt% REPS-L01 showed a partially disordered network structure, while the emulsion gel in Figure 8C,D showed a more ordered and compact network structure, which is consistent with the results of frequency scanning in Figure 7. There were more pores in the emulsion gel than in the REPS-L01 solution, which may be caused by the volatilization of *n*-hexane during lyophilization. The results indicated that the addition of *n*-hexane could fill the pores in the structure of REPS-L01, and the modulus and viscoelasticity of REPS-L01 were improved.

Under this ratio of emulsification, the interaction between *n*-hexane and REPS-L01 is similar to the interaction between polysaccharides and small-molecule emulsifiers [64]. Solvent *n*-hexane is insoluble in water as a non-polar molecule, so it exhibits hydrophobic properties similar to that of a small-molecule emulsifier, and could form molecular aggregates around the hydrophobic side chain of polysaccharide molecules, increasing the stability of polysaccharide conformation, and thus forming a strong gel. The gel network formed by the interaction of REPS-L01 with *n*-hexane has possible functions in drug transport systems [7]. However, further tests on the mechanical properties and thermal stability of the emulsion gel are required.

## 4. Conclusions

Based on the above studies, *Rhizobium tropici* L01 is a highly efficient polysaccharide producer, capable of producing up to 22.8 g/L REPS-L01 with simple medium components, and has good prospects in industrial production. Similar to other rhizobial exopolysaccharides, the composition of REPS-L01 contained common units of glucose and galactose, carrying pyruvate, acetate, and succinate groups. However, the unique molar ratio of 2.95:1 (glucose: galactose) makes it a novel rhizobial exopolysaccharide. REPS-L01 showed excellent rheological properties and thermal stability. It had excellent emulsifying activity with soybean oil and some alkanes, and especially with *n*-hexane, with which it formed a dense emulsion gel. The novel polysaccharide RPS-L01 exhibited excellent resistance to metal ions, stability across a broad pH range, and distinctive thickening and emulsifying properties. These characteristics render it a promising candidate as an emulsion stabilizer in the food, cosmetic, and other daily chemical industries. Future studies will focus on elucidating the mechanisms by which REPS-L01 forms an emulsion gel with solvents, which will help to explore the potential of REPS-L01 in nanomaterials and medicine. We will also try to scale up the production of REPS-L01 to improve its commercial potential.

## Figures and Tables

**Table 1:** Composition of the brine water.

Composition (mg/L)			**Total Salinity (mg/L)**
NaCl	CaCl_2_	MgCl_2_	
1516.6	180.1	303.3	2000
3791.5	450.2	758.3	5000
7582.9	900.5	1516.6	10,000
15,165.8	1801.0	3033.2	20,000

**Table 2:** Chemical composition of REPS-L01.

Parameters	REPS-L01
Total carbohydrate (%)	83.03
Uronic acid (%)	0
Protein (%)	0.68
Molar mass moments (g/mol)	
M_w_^a^	3.04 × 10^6^
M_n_^b^	2.63 × 10^6^
Dispersity index	1.155
Monosaccharide content (molar ratio, %)	
Glucose	74.7
Galactose	25.3
Substituent content (mg/g)	
Pyruvic acid	66.1
Succinic acid	10.2
Acetic acid	28.3

^a^ M_w_ and ^b^ M_n_ are weight-average molecular weight and number-average molecular weight, respectively.

## Data Availability

The original contributions presented in this study are included in the article/Supplementary Materials. Further inquiries can be directed to the corresponding author.

## Supplementary Materials

The following are available online at https://www.mdpi.com/article/10.3390/polym17050592/s1, Figure S1: GPC-SEC chromatogram for REPS-L01; Figure S2: The results of REPS-L01 monosaccharide composition analysis; Figure S3: The results of standard sample monosaccharide composition analysis.

## Abbreviations

REPS-L01: exopolysaccharide from the strain *Rhizobium* sp. L01; EPS, exopolysaccharide; REPS, rhizobial exopolysaccharide; NCBI, National Center for Biotechnology Information; HPLC, high-performance liquid chromatography; FTIR, Fourier-transform infrared; E_24_, emulsification index; SEM, scanning electron microscopy; SRA, sequence read archive; Mw, weight-average molar weight; Mn, number-average molar weight; TGA, thermal gravimetric analysis; DTG, differential thermal gravimetry; G′, elastic modulus; G″, viscous modulus.
