# Supplementary material for: Characterization and Rheological Properties of a New Exopolysaccharide Overproduced by Rhizobium sp. L01
Source: Polymers (Basel). 2025 Feb 23;17(5):592. doi: 10.3390/polym17050592 (PMC11902619; doi:10.3390/polym17050592)
Supplement: Supplementary file 1 [file polymers-17-00592-s001.zip › polymers-3479878-supplementary.pdf]

Supporting Information

Characterization and Rheological Properties of a New Exopolysaccharide Overproduced by *Rhizobium* sp. L01

Haolin Huang [1], Yaolan Wen [1], Zhuangzhuang Li [2], Biao Wang [2] and Shuang Li [1,*]

[1] College of Biotechnology and Pharmaceutical Engineering, Nanjing Tech University, Nanjing 211816, China; 202262118011@njtech.edu.cn (H.H.); 202261218125@njtech.edu.cn (Y.W.)
[2] Petroleum Engineering Technology Research Institute of Sinopec Jiangsu Oilfield Company, 188 Weiyang Road, Yangzhou 225000, China; lizz.jsyt@sinopec.com (Z.L.); wangbiao.jsyt@sinopec.com (B.W.)
* Correspondence: lishuang@njtech.edu.cn; Tel./Fax: 86-25-58139942

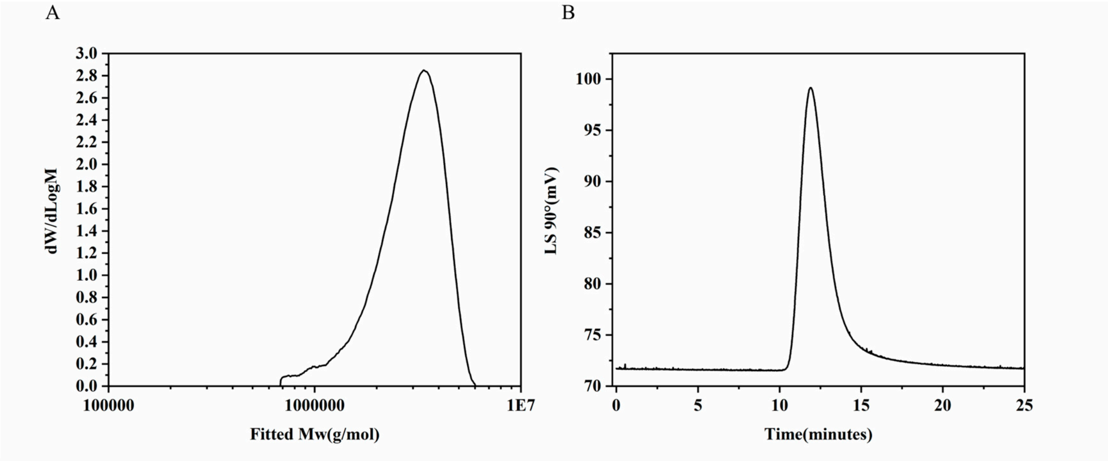

Fig. S1 GPC-SEC chromatogram for REPS-L01

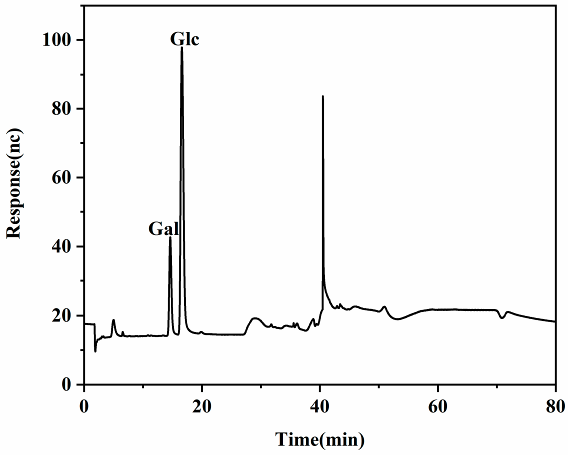

Fig. S2 The results of REPS-L01 monosaccharide composition analysis

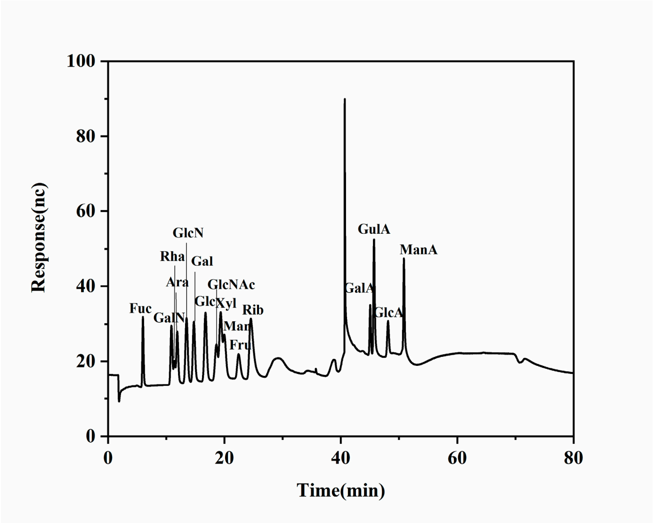

Fig. S3 The results of standard sample monosaccharide composition analysis
